# Nicotinamide improves in vitro lens regeneration in a mouse capsular bag model

**DOI:** 10.1186/s13287-022-02862-8

**Published:** 2022-05-12

**Authors:** Xiaomin Liu, Qingjun Zhou, Yusen Huang, Zheng Fan, Haoyun Duan, Menghan Wang, Zongyi Li, Lixin Xie

**Affiliations:** 1 State Key Laboratory Cultivation Base, Shandong Provincial Key Laboratory of Ophthalmology, Eye Institute of Shandong First Medical University, Qingdao, 26600 China; 2 Qingdao Eye Hospital of Shandong First Medical University, Qingdao, 26600 China; 3 Qingdao University Medical College, Qingdao, 26600 China

**Keywords:** Lens regeneration, Capsular bag culture, Nicotinamide, Differentiation, Transparency

## Abstract

**Background:**

Mammalian lens regeneration holds great potential as a cataract therapy. However, the mechanism of mammalian lens regeneration is unclear, and the methods for optimization remain in question.

**Methods:**

We developed an in vitro lens regeneration model using mouse capsular bag culture and improved the transparency of the regenerated lens using nicotinamide (NAM). We used D4476 and SSTC3 as a casein kinase 1A inhibitor and agonist, respectively. The expression of lens-specific markers was examined by real-time PCR, immunostaining, and western blotting. The structure of the in vitro regenerated lens was investigated using 3,3′-dihexyloxacarbocyanine iodide (DiOC6) and methylene blue staining, terminal deoxynucleotidyl transferase dUTP nick end labeling (TUNEL), and transmission electron microscopy.

**Results:**

The in vitro lens regeneration model was developed to mimic the process of in vivo mammalian lens regeneration in a mouse capsular bag culture. In the early stage, the remanent lens epithelial cells proliferated across the posterior capsule and differentiated into lens fiber cells (LFCs). The regenerated lenses appeared opaque after 28 days; however, NAM treatment effectively maintained the transparency of the regenerated lens. We demonstrated that NAM maintained lens epithelial cell survival, promoted the differentiation and regular cellular arrangement of LFCs, and reduced lens-related cell apoptosis. Mechanistically, NAM enhanced the differentiation and transparency of regenerative lenses partly by inhibiting casein kinase 1A activity.

**Conclusion:**

This study provides a new in vitro model for regeneration study and demonstrates the potential of NAM in in vitro mammalian lens regeneration.

**Supplementary Information:**

The online version contains supplementary material available at 10.1186/s13287-022-02862-8.

## Introduction

Cataract is a leading cause of blindness in the world [1], and its common clinical treatment is surgical lens extraction and implantation of an artificial intraocular lens (IOL). However, the application of an IOL, especially in pediatric patients, has many limitations, such as posterior capsular opacification (PCO), inflammation, available choices of intraocular lens power and type, and the risk of secondary glaucoma and even secondary blindness [2–5]. One potential alternative to IOL application in cataract patients is lens regeneration, as mammalian lens epithelial cells (LECs) can proliferate and differentiate after lens injury [6–8]. Very recently, Lin et al. reported a novel surgical technique for minimally invasive lens-content removal surgery (MILS), which preserved the lens capsule and the LECs to an extent that permitted the partial functional restoration and in situ regeneration of the lens in human infants [6]. However, this method is not applicable to a large number of older patients, and the long-term clinical efficacy and safety of the procedure need further follow-up for verification. In addition, achieving a lens with intact biological functions and optical properties still remains a major bottleneck in in situ lens regeneration.

A few studies have examined the molecular mechanisms of mammalian lens regeneration, but most have been limited to histological observations [9]. The use of an in vitro model of mammalian lens regeneration would therefore be beneficial for understanding the signaling pathways involved and for optimizing regeneration strategies. However, in vitro lens regeneration remains at the stage of cultivating stem cells to produce tissues with lens-like morphologies and structures used mainly in cataract-related research, as the clinical application and transplantation of regenerated lenses are limited by the need for a cultivation period and by the high cost [10].

The experimental systems most frequently used in in vitro lens cultivation are the capsular bag model and lens epithelial explant system, but these have mostly been used to study the mechanism of PCO [11, 12]. In the present study, we established an in vitro capsular bag cultivation model supported by low-melting-point (LMP)-agarose gel, which maintains the natural capsule contours and recapitulates the process of mammalian lens regeneration in vivo. We also tested the efficacy of using nicotinamide (NAM), a component of the vitamin B3 family, that is extensively applied in the in vitro culture of organoids [13–16] and for the induction of differentiation in stem cells [17–20]. Here, we demonstrated that NAM retained transparency of the regenerating lens in vitro, maintained the survival of a greater number of LECs, and promoted LECs differentiation and the regular cellular arrangement of fiber cells while reducing apoptosis of lens-related cells. Our findings identified an in vitro model that can be used to improve mammalian lens regeneration and aid in mechanistic studies. Our study also demonstrated a potential beneficial effect of using NAM for the in vitro regeneration of a transparent lens.

## Materials and methods

### Establishment of an in vitro mouse capsular bag model

Male C57BL/6J male mice (4–5 weeks old) purchased from the Weitonglihua Company (Beijing, China) were sacrificed by cervical dislocation, and the eyeballs were harvested. Under a stereo microscope, the cornea was cut along the limbus with ophthalmic scissors, the iris was separated, the suspensory ligament was disconnected, and the lens was removed. A small opening was then cut on the center of the anterior capsule to mark the front surface. The mixed tissues surrounding the lens were avoided when removing the lens.

A 2% (*w*/*v*) solution of low-melting-point (LMP)-agarose gel (Solarbio, A8350) was boiled and then cooled to 37 °C on a water bath to achieve a physiological temperature that would not inflict damage to the LECs on the capsule during the lens molding procedures. Each marked lens was positioned at the central portion of a fabricated disposable embedding mold. Subsequently, the 37 °C liquefied LMP-agarose gel solution was slowly dropped into the mold, avoiding bubble formation, until the liquid level crossed the zonular attachment position. The molds were then placed for 2 min at 4 °C to solidify the LMP-agarose gel. A 0.8-mm cross-shaped incision was introduced on the center of the anterior capsule, and the lens fiber mass was removed via hydrodissection. The remaining cortical fibers were completely removed.

The capsular bags were then incubated at 37 °C in a humidified atmosphere containing 5% CO_2_ and 95% air. The basal culture system was initiated with advanced Dulbecco’s modified Eagle’s medium (DMEM)/F12 (Gibco, 11330-032), supplemented with 1 × penicillin–streptomycin (Corning, cat#: 30-002-CI), 1 × GlutaMax (Gibco, cat#: 35050-061), 2% fetal bovine serum (FBS, Gibco, cat#: 10099-141C), 1 × B-27 (Gibco, cat#: 17504-044), and 5 ng/mL bFGF (RD, 233-FB), and cultured for 1 week. In the following week, the 5 ng/mL bFGF was changed to 150 ng/mL bFGF. 10 mM NAM (Sigma) was added since 1, 7 and 14 days after the in vitro culture.

The mechanism of NAM was explored by adding five molecules: SB431542 (1 μM; Calbiochem, cat#: 616461), Sirtnol (10 μM; Selleck, cat#: S2804), nicotinamide adenine dinucleotide (NAD+, 800 nM; Sigma, cat#: 481911), CKi (10 μM; D4476, MCE, cat#: HY-10324), and SSTC3 (10 μM; MCE, cat#: HY-120675).

All animal experiments were approved by the ethics committee of Shandong Eye Institute and were conducted in accordance with the Association for Research in Vision and Ophthalmology Statement for the Use of Animals in Ophthalmic and Vision Research.

### Immunofluorescence staining

The whole mounts of regenerated mouse lenses supported by LMP-agarose gel and of mouse eyeballs were embedded in TissueTek OCT compound (Sakura Finetek, Tokyo, Japan). Frozen sections (7 μm) were fixed in 4% paraformaldehyde (PFA) for 15 min, permeabilized with 0.5% TritonX-100 for 15 min for detection of nucleus-specific antibodies or with 0.1% TritonX-100 for 5 min for cytoplasmic-specific antibodies, and then blocked with 5% BSA for 1 h at room temperature. The sections were incubated overnight at 4 °C with antibodies against PAX6 (Proteintech Group, Inc Rosement, IL 60018, USA), PROX1 (Abcam, Cambridge, UK), αA-crystallin (Abcam, Cambridge, UK), αB-crystallin (Abcam, Cambridge, UK), β-crystallin (Abcam, Cambridge, UK), MIP (Santa, Cruz, CA, USA), and CK1A (Proteintech Group, Inc Rosement, IL 60018, USA) and subsequently incubated with fluorescein-conjugated secondary antibody (1:200; Beijing Zhongshan Jinqiao Biotechnology Co., Ltd., Beijing, China) for 1 h, followed by staining with 4′,6-diamidino-2-phenylindole (DAPI; Solarbio, Beijing, China) for 5 min. All stained sections were observed and captured using an Echo Revolve microscope (Echo Laboratories, San Diego, California).

### Terminal deoxynucleotidyl transferase dUTP nick end labeling (TUNEL) assay

Apoptosis was measured by TUNEL assays using an In Situ Cell Death Detection kit (Roche Diagnostics GmbH, Mannheim, Germany). Briefly, the regenerated lens sections were fixed with 4% PFA for 15 min and then permeabilized in 0.1% sodium citrate containing 0.1% Triton X-100 for 2 min on ice. The sections were then incubated with TUNEL reaction solution at 37 °C for 1 h and observed using an Echo Revolve microscope (Echo Laboratories, San Diego, California). Images were analyzed using ImageJ software, and the percentage of apoptotic cells was counted as the number of positive apoptotic cells in the center area of each regenerated lens divided by the total number of cells in the center area × 100. Each group was replicated at least three times.

### Histological evaluation

Mouse lens tissue sections were prepared as described above. The frozen sections were stained with hematoxylin–eosin (H&E) and 1% methylene blue by conventional methods and observed with a Nikon N1-U microscope. The membranes in the sections were also stained with DiOC6 (5 ug/mL) for analysis of the cellular arrangement and observed with an Echo Revolve microscope (Echo Laboratories, San Diego, California).

### Transmission electronic microscopy (TEM)

Whole regenerated lenses supported by LMP-agarose gel and normal mouse lenses were fixed overnight at 4 °C with 2.5% glutaraldehyde (Solarbio, Beijing, China). The samples were washed with phosphoric acid rinse solution (0.1 M, pH 7.0), postfixed with 1% OsO4, and embedded in Epon812 resin. The regenerated lenses were sectioned with a Reichert-Jung Ultracut E ultramicrotome. The sections (70 nm) were stained with uranyl acetate and alkaline lead citrate, and images of the sections were captured with a transmission electronic microscope (JEM1200, JEOL, Japan).

### Quantitative real‐time polymerase chain reaction (qPCR)

The total RNA of regenerated lenses was extracted using the TransZolTM Up Plus RNA kit (TransGen Biotech, Beijing, China), according to the manufacturer’s guidelines, and then, cDNA was synthesized using the Prime Script^™^ RT reagent kit (Toyobo, Tokyo, Japan). The cDNA was analyzed with the ChamQ Universal SYBR qPCR Master Mix (Vazyme Biotech Co., Ltd., Nanjing, China) with the primers shown in Table 1, using the Roter-Gene Q system (Qiagen, Valencia, CA).

**Table 1:** Primers used for qPCR

Gene	Forward primer 5′–3′	Reverse primer 5′–3′
*m-pax6*	GCCCTCACCAACACGTACAGT	ATCATAACTCCGCCCATTCACT
*m-prox1*	ACCTTATTCAGGAAGCGCAATG	TGCGAGGTAATGCATCTGTTG
*m-foxe3*	TCATACATCGCGCTCATTGC	ACCTTGACGAAACAGTCGTTGA
*m-cryaa*	ACGAGAGGCAGGATGACCAT	CCAAACCGGACTGGACCTT
*m-cryab*	TGACACCGGACTCTCAGAGATG	TGTTCGTCCTGGCGTTCTTC
*m-cryba2*	CAGTGGCCACCACAGCAA	CCCATGGAAGGCAGTGATG
*m-crybb1*	CTGCCTTCCGTGGAGAGATG	CCCCTTCGAACAGGCAGAT
*m-crybb2*	GCTCTCTGAGGCCCATCAAA	GCACGGAAGACACCTTTTCC
*m-crygd*	GCAGTGGATGGGTTTCAGTGA	TGGAATCGGTCCTGGAGAGA
*m-crygc*	CTACCAGGGCCACCAGTACTTC	TCCATCATGACACCTTTGTGATCT
*m-mip*	GAGATCTTCTTGACGCTCCAGTTC	CATCCCCGCACCAGTGTAAT
*m-bfsp1*	ATTGCGTAACCTGCACCTTCA	AGGGACACTTGAGGAGCAGTCT
*m-bfsp2*	GCTGCTGCCCTCAGTGTAGAG	CAGGTTCTGCAGTTCCATGTCA
*m-lgsn*	CCCCGCACAGTTTTTTCAAG	GACAGCTCTGGCATGAGGACTA
*m-gapdh*	GCCACCCAGAAGACTGTGGAT	GGAAGGCCATGCCAGTGA

### Western blot analysis

Total protein was extracted using radioimmunoprecipitation assay (RIPA) buffer, and samples (20 μg) were run on 12.5% sodium dodecyl sulfate polyacrylamide gel electrophoresis (SDS-PAGE) gels and transferred to polyvinylidene fluoride (PVDF) membranes (Millipore, Billerica, MA, USA). The membranes were incubated with antibodies against CK1A (Proteintech Group, Inc Rosement, IL 60018, USA), αA-crystallin (Abcam, Cambridge, UK), β-crystallin (Abcam, Cambridge, UK), or MIP (Santa, Cruz, CA, USA), followed by incubation with horseradish peroxidase-conjugated secondary antibodies (Proteintech Group, Inc Rosement, IL 60018, USA). GAPDH (Kangchen, Shanghai, China) was detected as a loading control. The signal was detected by the ECL^™^ chemiluminescent system (Millipore, Billerica, MA, USA).

### Statistical analysis

All experiments were performed at least three times, and the data were expressed as mean ± standard deviation. Statistical analysis was conducted with GraphPad Prism8. Differences between the two groups were tested with an unpaired *t*-test, and more than two sets of data were compared using one-way analysis of variance (ANOVA). A value of *p* < 0.05 was considered statistically significant.

## Results

### Establishment and early observation of the in vitro mouse lens capsular bag culture model

**Fig. 1:**
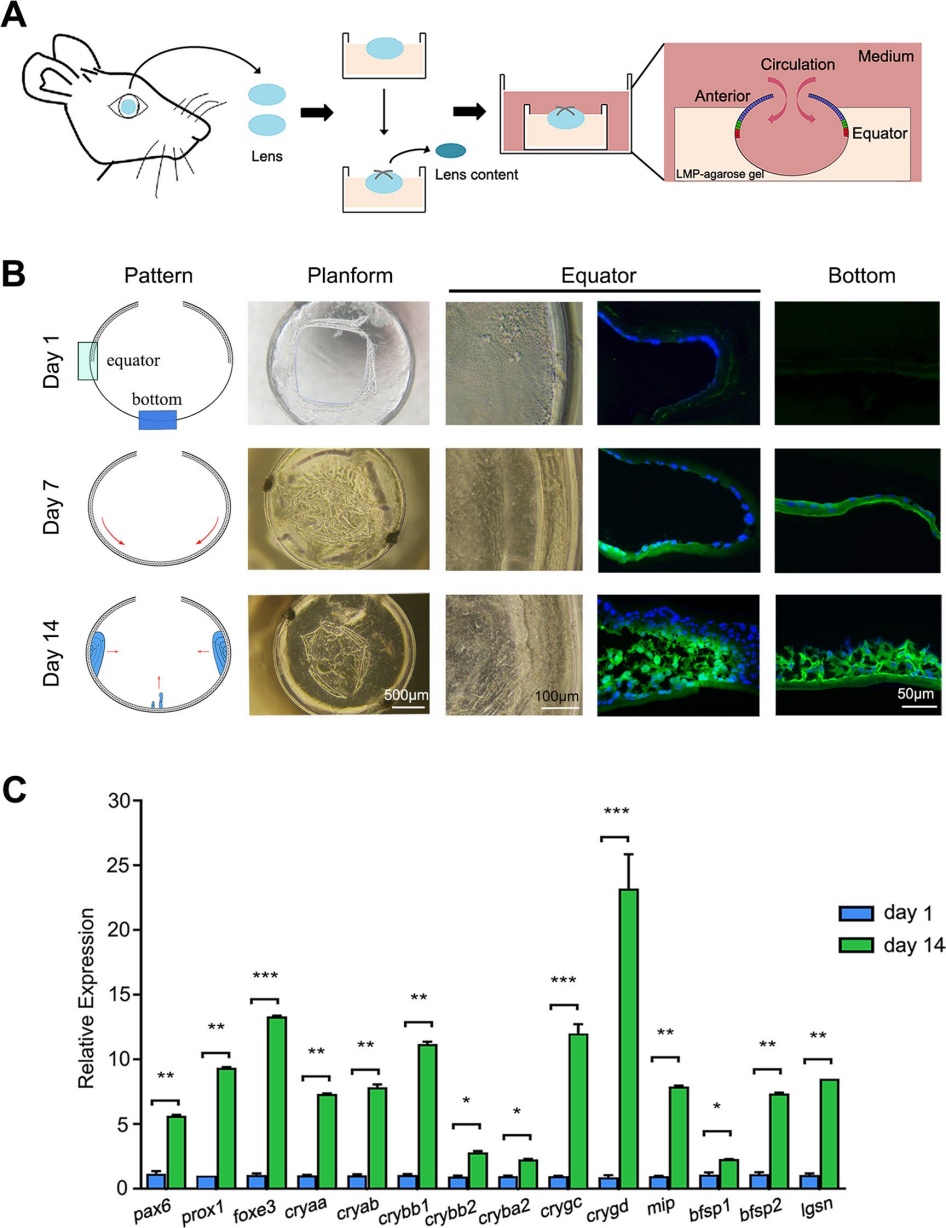
Regeneration and characterization of the in vitro cultured mouse lens by the capsular bag culture model. **A** Pattern of the establishment of an in vitro mouse lens capsular bag culture model. **B** The diagram on the left column shows the pattern of the lens regeneration; the progression of regeneration of the cultured lenses was followed by microscopy and by staining of β-crystallin (green) at days 1, 7, and 14. Nuclei were stained with DAPI (blue). **C** The mRNA levels of the LEC-related and LFC-related genes were assessed by qPCR 14 days after in vitro culture and compared with the levels at day 1 (*n* = 4). Data are presented as mean ± SD. Unpaired *t*-test. *p < 0.05, **p < 0.01, ***p < 0.001

The lenses were removed from the mice and fixed with LMP-agarose gel in a self-made mold. The lens content was then separated through a 0.8-mm cross-shaped incision from the center of the anterior capsule, with the LECs preserved. The lens capsular bag was cultured for in vitro lens regeneration (Fig. 1A). The transparent LMP-agarose gel allowed convenient observation of the three-dimensional spherical structure of the lens, and the cells were clearly visible by microscopy (Fig. 1B).

The progression of regeneration of the cultured lenses was determined by analyzing the expression of β-crystallin, an early differentiation marker of lens fiber cells (LFCs), at different times by immunofluorescence. The lens regeneration pattern shown in the diagram on the left column reflected the β-crystallin expression (Fig. 1B), which was negative in the anterior capsule and the equatorial part of the lens immediately after the lens-content removal, but then slightly increased in the posterior capsule at 7 days. Subsequently, β-crystallin–positive cells proliferated and elongated toward the center of the cultured lens and accumulated in the posterior capsule within 14 days (the right two columns in Fig. 1B). This pattern was similar to the in vivo pattern of mammalian lens regeneration [21]. The qPCR data showed that the expression of the LEC and LFC genes significantly increased 14 days after initiation of the in vitro culture (Fig. 1C). These findings suggested that the preserved LECs proliferated and differentiated into LFCs in vitro in our three-dimensional culture model of the lens capsular bag and mimicked the in vivo process of mammalian lens regeneration*.*

### NAM promoted the transparency and differentiation of the regenerated lens

**Fig. 2:**
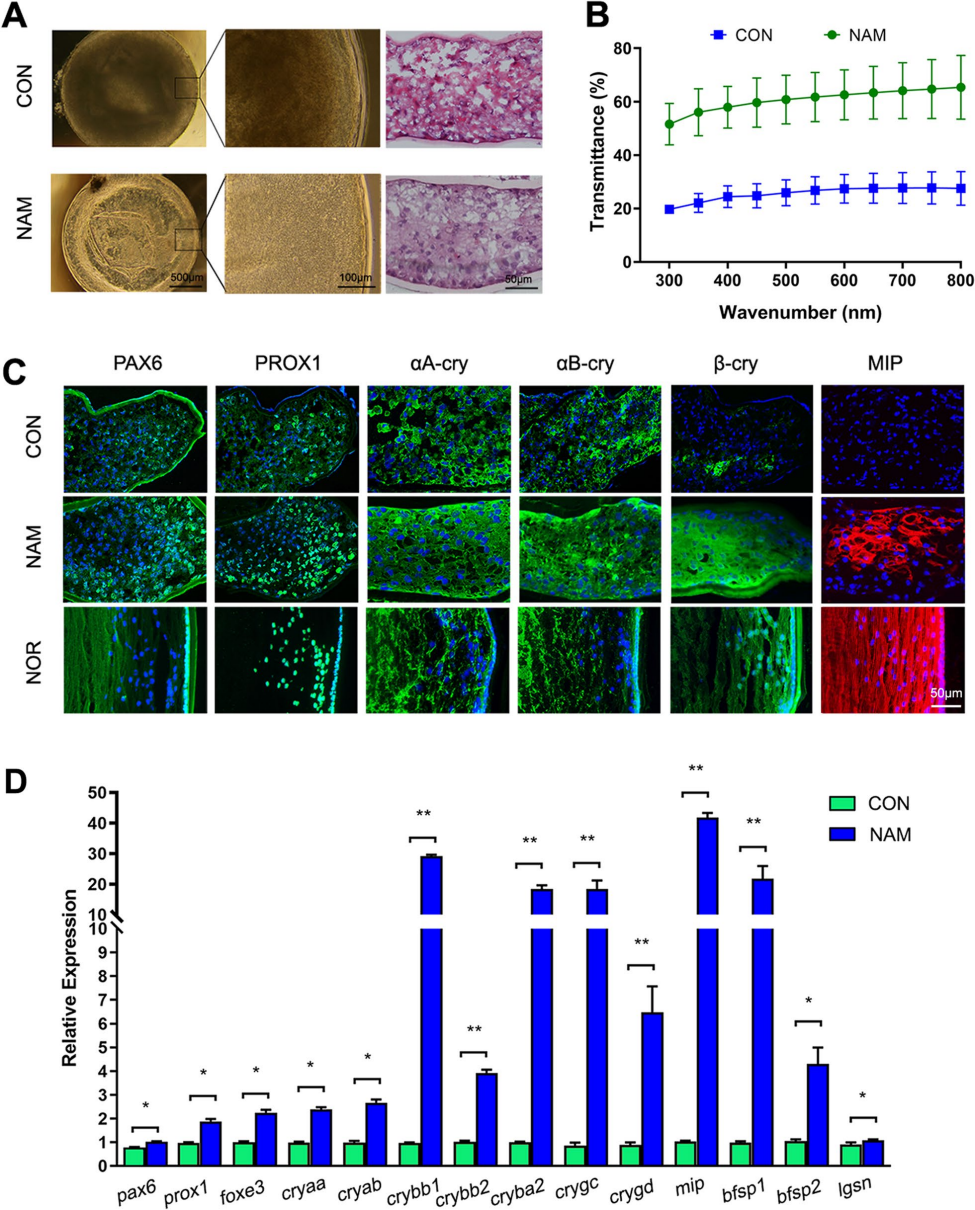
Effect of NAM on the transparency and differentiation of the regenerated lens after 28 days of follow-up. **A** General images of the regenerated lenses with or without NAM treatment and H&E staining. **B** Light transmittance of the two lens groups at different wavelengths. Data are means ± SD of 4 regenerative lenses per group. **C** Representative immunofluorescence staining showing the expression of the LEC markers PAX6 (green), αA-crystallin (green), and αB-crystallin (green), and the LFC markers PROX1 (green), β-crystallin (green), and MIP (red) in the NAM-treated (NAM) and untreated control (CON) lens groups. A normal lens (NOR) was used as a control. Nuclei were stained with DAPI (blue). **D** The qPCR results evaluating the genes of LECs and LFCs in the untreated control (CON) and NAM-treated groups (*n* = 4). Data are presented as mean ± SD. Unpaired *t*-test. *p < 0.05, **p < 0.01

In the long-term in vitro culture, the regenerated lenses appeared opaque and decreased expression of lens-related genes after 28 days (Fig. 2A and Additional file 1: Fig. S1). The transparency of the regenerated lenses was improved following the introduction of NAM since 1, 7 or 14 days, but the application of NAM in the late stage simultaneously maintained the proliferation and transparency of regenerated lenses (Additional file 2: Fig. S2). NAM has been reported to induce the differentiation of stem cells and is extensively used for the in vitro culture of organoids [13–20]. NAM maintained the transparency of the regenerated lenses, which exhibited a better cellular arrangement than the untreated control lenses, as determined by H&E staining (Fig. 2A). The light transmittance was also better in the NAM group than in the CON group (Fig. 2B).

The effect of NAM on the differentiation of the regenerated lenses was determined by immunostaining for the expression of the lens-related genes, including the LEC markers PAX6, αA-crystallin, and αB-crystallin [22, 23], the regulation marker of LFC differentiation PROX1 [24], and the LFC markers β-crystallin and MIP. The NAM treatment increased the expression of these markers in the regenerated lenses after 28 days (Fig. 2C). The NAM treatment also induced PAX6 and PROX1 expression in the peri-equatorial part of the lens and β-crystallin and MIP near the central part, in agreement with the patterns seen in normal lenses (Fig. 2C). The qPCR results also verified that NAM increased the expression of LEC-related and LFC-related genes after 28 days (Fig. 2D). These findings indicated that NAM improved the survival of LECs and promoted the differentiation of LECs to maintain the transparency of in vitro regenerated lens.

### NAM improved the structure of the regenerated lens

**Fig. 3:**
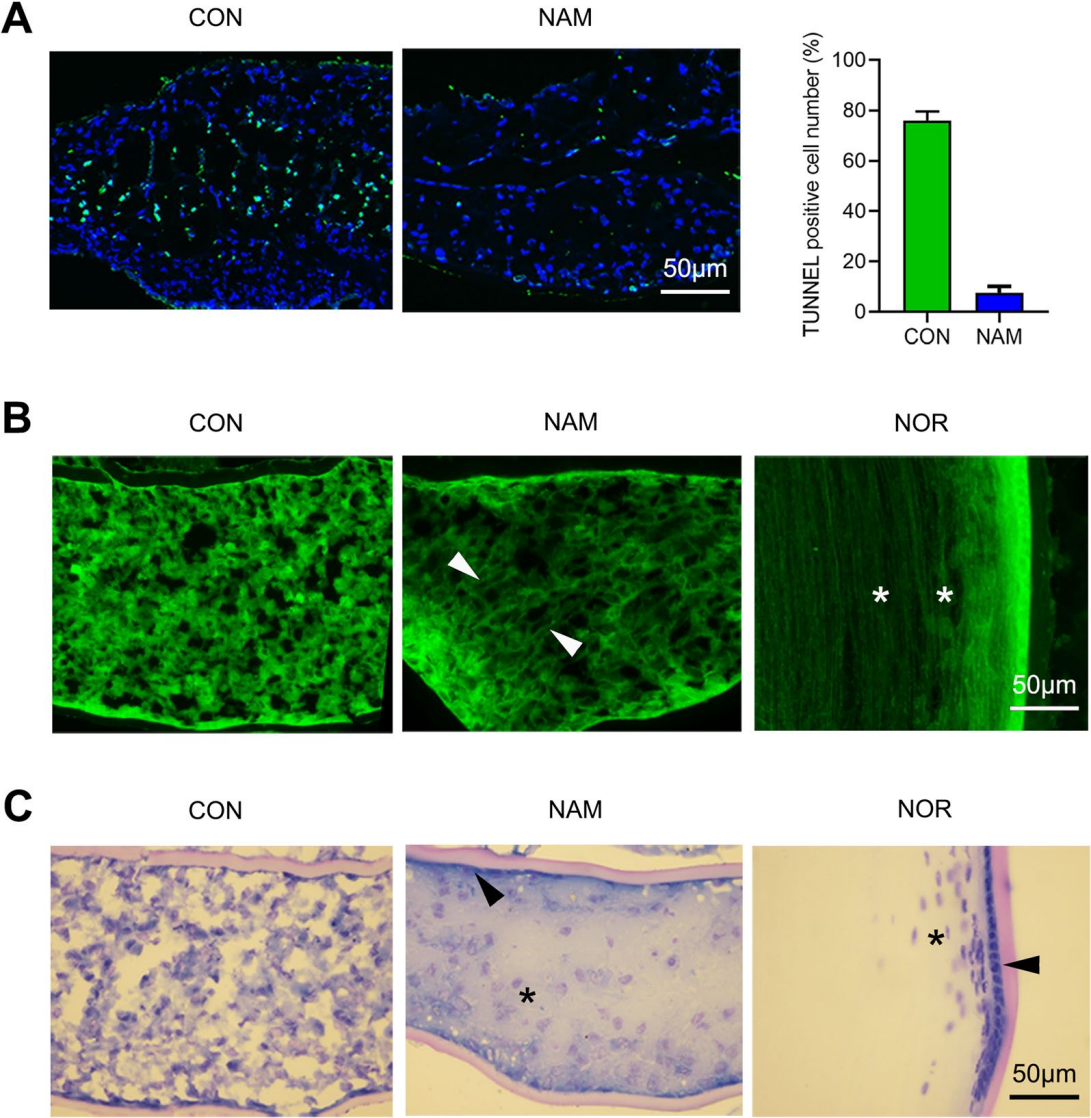
Effect of NAM on the structure of the regenerated lens. **A** TUNEL staining (green) showing cell apoptosis in the untreated control (CON) group and the NAM-treated (NAM) group. Nuclei were stained with DAPI (blue). **B** DiOC6 staining showing the cellular arrangement of the regenerated lens in the CON and NAM groups. White arrowheads indicate the cellular arrangement of the regenerated lenses treated with NAM, and white asterisks indicate the cellular arrangement of normal lenses. **C** The nuclear features (blue) in the two groups revealed by methylene blue staining. Black arrowheads indicate the nuclear features of the LECs in the capsule, and black asterisks indicate the nuclear features of the LFCs

The beneficial effect of NAM on the long-term transparency of regenerated lenses was further determined by examining the structure of the regenerated lenses after 28 days. TUNEL staining revealed many apoptotic cells in the center area of the lens in the untreated control group, but fewer apoptotic cells in the NAM-treated group (76 ± 3.6% vs. 7.6 ± 2.5%, *p* < 0.01) (Fig. 3A). DiOC6 staining showed that part of the LFCs in the NAM-treated regenerated lenses showed an elongated cellular arrangement that was similar to the LFC pattern seen in the normal lens (Fig. 3B). However, the DiOC6 staining in the untreated control group revealed abnormal cells containing many particles (Fig. 3B). Methylene blue staining performed to evaluate the cell nuclear features revealed LEC and LFC properties similar to those in the normal lens in the NAM-treated regenerated lenses, with blue staining in the cell peripheral cells and faded blue in the center cells (Fig. 3C). However, lenses in the untreated control group showed extensive blue staining, and the outlines of the nuclei were difficult to discern (Fig. 3C).

**Fig. 4:**
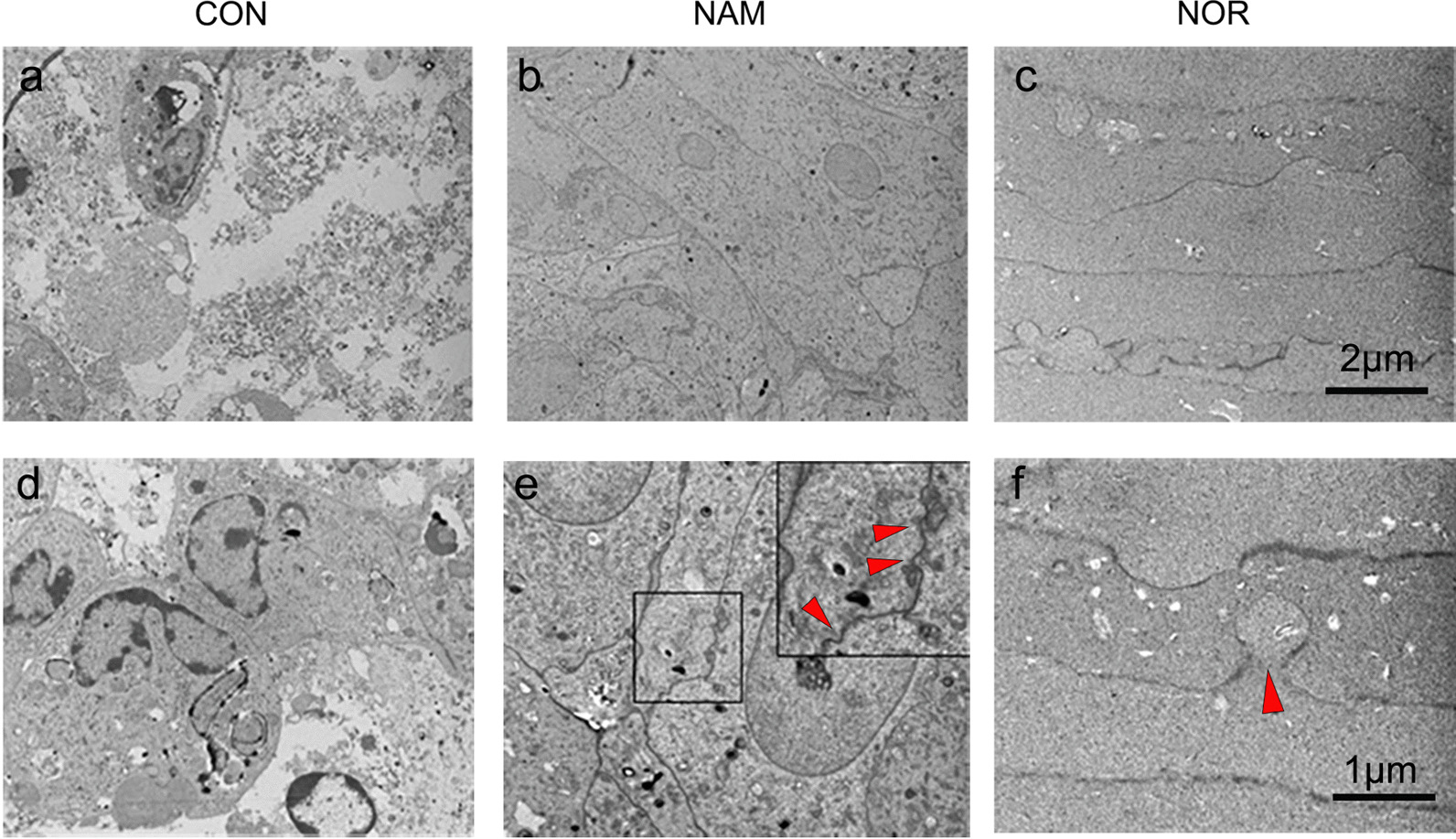
Effect of NAM on the ultrastructure of the regenerated lens. Transmission electron microscopy (TEM) showing the cellular degeneration and arrangement between untreated control (CON,** a** and** d**), NAM-treated regenerated lenses (NAM,** b** and** e**) and normal lenses (NOR,** c** and** f**). Red arrowheads indicate membrane interdigitations of the LFCs. Scale bar:** a**–**c** 2 μM,** d**–**f** 1 μM

The TEM images confirmed that the cells in the untreated control group had experienced cellular degeneration (Fig. 4a, d), whereas the cells in the NAM-treated group had become elongated and closely packed (Fig. 4b), in a pattern similar to that of normal LFCs (Fig. 4c). The LFCs in the NAM-treated lens also exhibited membrane interdigitations typical of normal LFCs (Fig. 4e, f). These findings suggested that NAM maintained cellular integrity and promoted an accurate ordering of fiber differentiation.

### NAM promoted differentiation of the regenerated lens through CK1A inhibition

**Fig. 5:**
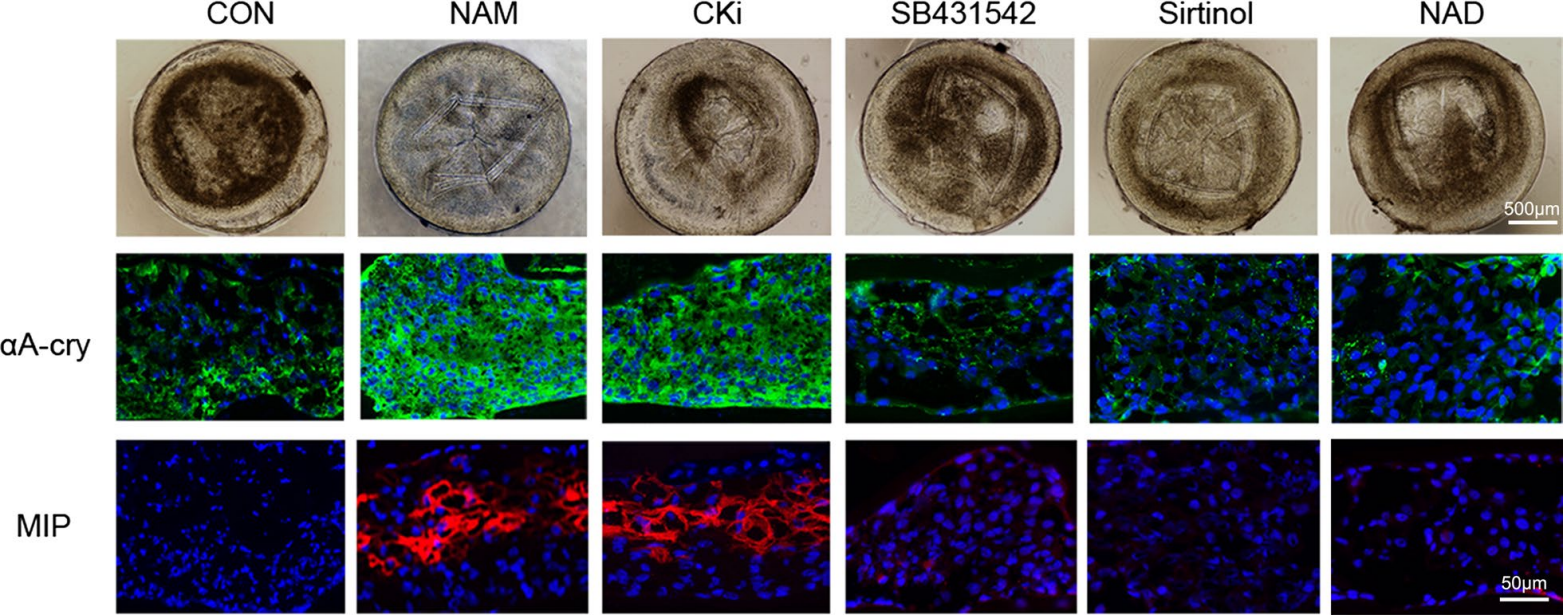
Effects of the activator/inhibitor molecules on the differentiation of regenerated lenses. Effects of four specific activators/inhibitors, including inhibitors of SIRT1 (Sirtnol), TGFβ (SB431542), casein kinase 1A (CKi) and NAD+ (NAD), representing different signaling pathways of NAM on the transparency (*n* = 3) and immunofluorescence staining of CK1A1 (green) and MIP (red) in regenerated lenses. Nuclei were stained with DAPI (blue)

Considering the known roles of NAM [19, 20, 25–30], the effects of NAM were mimicked by treating the in vitro cultured lenses with several specific activators/inhibitors, including inhibitors of SIRT1 (Sirtnol), TGFβ (SB431542), casein kinase 1A (CKi) and NAD+ (NAD). Only the regenerated lens treated with CKi appeared relatively transparent (Fig. 5).

**Fig. 6:**
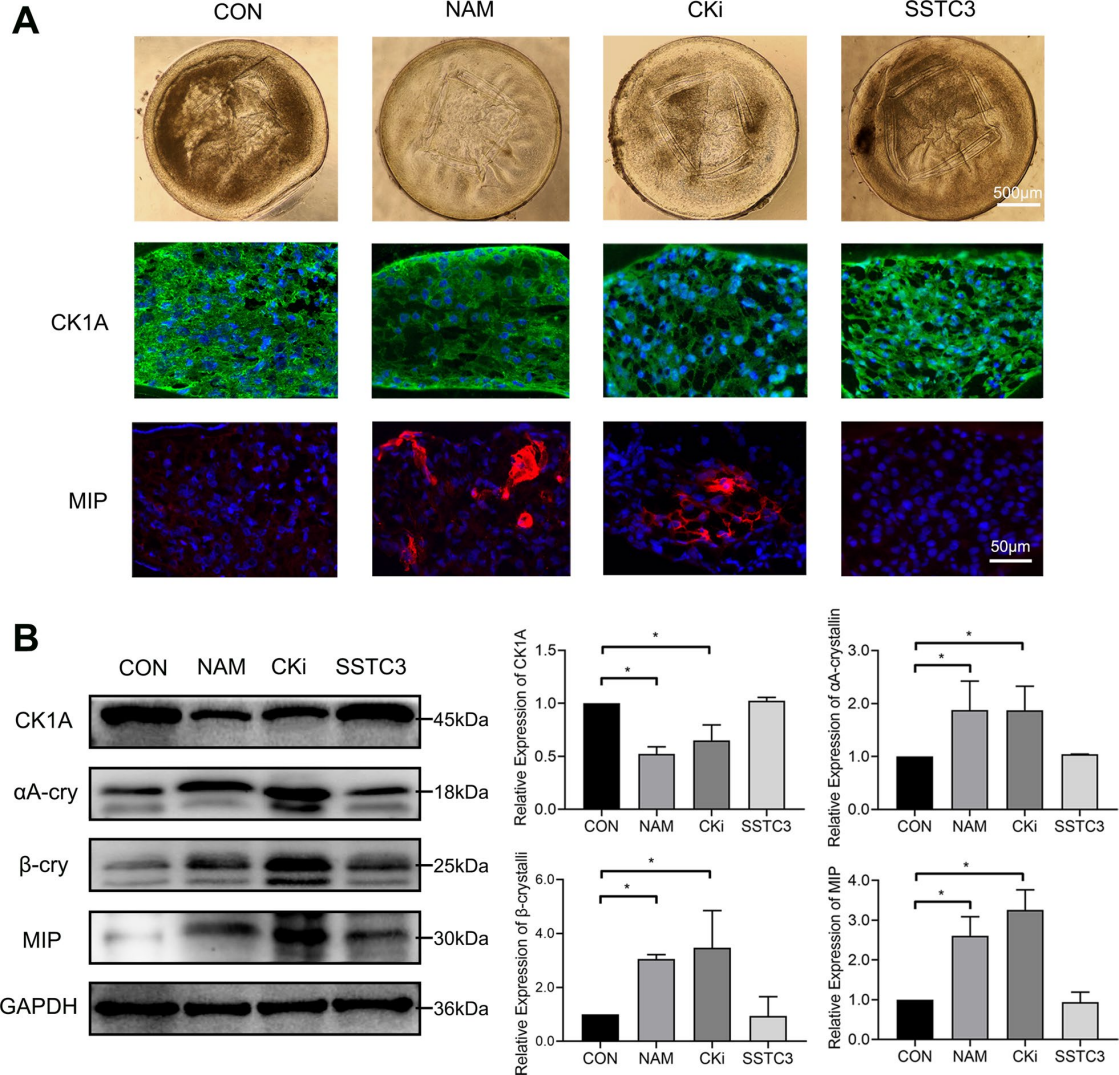
Effect of CK1A on the differentiation of regenerated lens. **A** The transparency of the regenerated lenses following NAM, CKi, or SSTC3 treatment versus untreated controls (CON). Immunofluorescence results showing CK1A (green) and MIP (red) expression in the CON, NAM, CKi, or SSTC3 groups. Nuclei were stained with DAPI (blue). **B** The representative bands of CK1A, αA-cry (αA-crystallin), β-cry (β-crystallin), and MIP in the CON, NAM, CKi, and SSTC3 groups and the relative CK1A, αA-crystallin, β-crystallin, and MIP protein levels, with GAPDH as the internal control. *n* = 4. One-way ANOVA. *p < 0.05

We further investigated whether NAM promotes lens regeneration through CK1A signaling by treating the regenerated lenses with NAM, CKi, or the CK1A agonist SSTC3. Untreated lenses were used as a control group. Consistently, the regenerated lenses treated with CKi or NAM were transparent, while the lens treated with SSTC3 showed similar opacity to the untreated control lenses (Fig. 6A). Immunofluorescence results showed high expression of CK1A in the control and SSTC3 groups but decreased expression in the NAM and CKi groups (Fig. 6A). More MIP-positive cells were present in the NAM and CKi groups than in the CON and SSTC3 groups (Fig. 6A). The western blotting results further verified the higher expression of CK1A in the CON and SSTC3 groups than in the NAM and CKi groups, as well as the higher expression of LEC- and LFC-related markers in the NAM and CKi groups than in the CON and SSTC3 groups (Fig. 6B). Altogether, these results imply that NAM may promote cell differentiation and transparency in the in vitro regenerated lens through CK1A inhibition.

## Discussion

Lens regeneration after cataract surgery is a particularly attractive avenue of research. Lin et al. developed the MILS procedure to utilize endogenous LECs for lens regeneration in pediatric patients and were the first to bring lens regeneration into the clinic [6]. However, this strategy continues to have many issues, such as aging, scarring of the anterior capsule, a long regeneration period, suboptimal lens transparency, and poor efficacy of lens regeneration [10, 31–34]. Further exploration and study are therefore needed to determine how best to optimize mammalian lens regeneration and how to change the tendency of the LFCs to show disordered growth. In the present study, we established an in vitro lens regeneration model that recapitulates the pattern of mammalian lens regeneration in vivo*.* Importantly, we found that NAM promoted the differentiation and regular arrangement of LFCs and maintained lens transparency throughout the lengthy in vitro culture period.

Two in vitro experimental lens culture systems, namely a capsular bag model and a lens epithelial explant system, have been developed to study the mechanisms of PCO [11, 12]. By contrast, no in vitro model has yet been developed for mammalian lens regeneration. Human-induced pluripotent stem cells (hiPSCs) and human embryonic stem cells (hESCs) have been induced to form lens cells and the transparent lentoid [35, 36], but the heterogeneity, size, lengthy cultivation period, and high cost have limited the clinical application of this approach. The present study used LMP-agarose gel to maintain the shape of the capsular bag, thereby avoiding changes in the mechanical environment of the capsular bag. The remanent LECs then proliferated along the anterior and posterior capsule and differentiated into fiber cells, recapitulating the process of mammalian lens regeneration in vivo*.* This in vitro model provides a new paradigm for studying the mechanism of mammalian lens regeneration.

One of the main challenges in mammalian lens regeneration is maintaining transparency, which involves two main processes: the programmed removal of organelles and the correct assembly of epithelial and fibrous cells [4]. The LEC pool plays an important role in the maintenance of lens function [6], and a confluent LEC monolayer over essentially the entire capsule is beneficial for regular alignment of regenerating LFCs [21]. On the contrary, the loss of LECs leads to abnormal cellular morphology and cellular degeneration [37]. Here, we demonstrated that NAM improved the maintenance of the LECs and promoted LFCs differentiation and regular arrangement, thereby maintaining the transparency of the in vitro regenerated lens throughout the long-term culture period. The effects of NAM on the in vivo lens regeneration in other mammals, such as rabbits, need further study.

Unlike the case for Wolffian lens regeneration and cornea-lens regeneration, little is known about the signaling pathways involved in mammalian lens regeneration. The present study demonstrated that NAM treatment reduced the expression of CK1A, a molecule that regulates many signaling pathways [38], including the Wnt, Hedgehog, and autophagy pathways that play important roles in lens regeneration and development [39, 40]. Here, we found that treatment of regenerating lenses with a CK1A inhibitor induced the regeneration of a relatively transparent lens, similar to that induced by NAM. NAM has been reported to promote the differentiation of hiPSCs and hESCs through CK1A inhibition [19, 20], further suggesting the involvement of CK1A in mammalian lens regeneration. Although CKi treatment improves the transparency of the regenerated lenses, these lenses were still worse than the ones in NAM group. These data suggested the CK1A signaling partly explained the mechanism of NAM on the lens regeneration. In addition, NAM is best known in enhancing cell survival, improving reprogramming, facilitating differentiation, regulating DNA repair, suppressing apoptosis and promoting the development and self-renewal, which is a multifunctional factor [20, 41–43]. Therefore, we would repeat the in vitro lenses regeneration for RNAseq analysis and specific transgenic mouse models for better understanding of the mammalian lens regeneration and NAM functions in the following study.

## Conclusion

We established an in vitro lens regeneration model that recapitulates the in vivo process of mammalian lens regeneration to explore and optimize in vitro mammalian lens regeneration. We demonstrated that NAM induced the in vitro regeneration of a transparent lens, suggesting the potential use of NAM in clinical lens regeneration.

## Supplementary Information

**Additional file 1: Fig. S1.** The expression of lens-related genes of a complete development process in the in vitro lens regeneration model. The mRNA levels of the LEC-related and LFC-related genes were assessed by qRT-PCR at day 1, day 14 and day 28 without any treatment.

**Additional file 2: Fig. S2.** The effect of NAM on the in vitro regenerated lens at different time point.

## Data Availability

All datasets generated for this study are included in the article.

## Abbreviations

NAM: Nicotinamide
DiOC6: 3,3′-Dihexyloxacarbocyanine iodides
TUNEL: Terminal deoxynucleotidyl transferase dUTP nick end labeling
LFCs: Lens fiber cells
IOL: Intraocular lens
PCO: Posterior capsular opacification
LECs: Lens epithelial cells
MILS: Minimally invasive lens-content removal surgery
LMP: Low-melting-point
DMEM: Dulbecco’s modified Eagle’s medium
FBS: Fetal bovine serum
qPCR: Quantitative real‐time polymerase chain reaction
RIPA: Radioimmunoprecipitation assay
SDS-PAGE: Sodium dodecyl sulfate polyacrylamide gel electrophoresis
PVDF: Polyvinylidene fluoride
TEM: Transmission electronic microscopy
hiPSCs: Human-induced pluripotent stem cells
hESCs: Human embryonic stem cells
