# Supplementary figures and images for: Nicotinamide improves in vitro lens regeneration in a mouse capsular bag model
Source: Stem Cell Res Ther. 2022 May 12;13:198. doi: 10.1186/s13287-022-02862-8 (PMC9102750; doi:10.1186/s13287-022-02862-8)

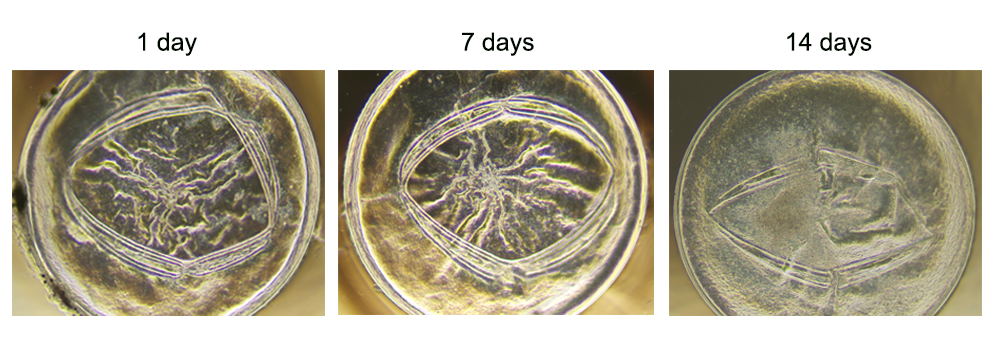

Supplement: Supplementary file 1 — Additional file 1: Fig. S1. The expression of lens-related genes of a complete development process in the in vitro lens regeneration model. The mRNA levels of the LEC-related and LFC-related genes were assessed by qRT-PCR at day 1, day 14 and day 28 without any treatment. [file 13287_2022_2862_MOESM1_ESM.tif]

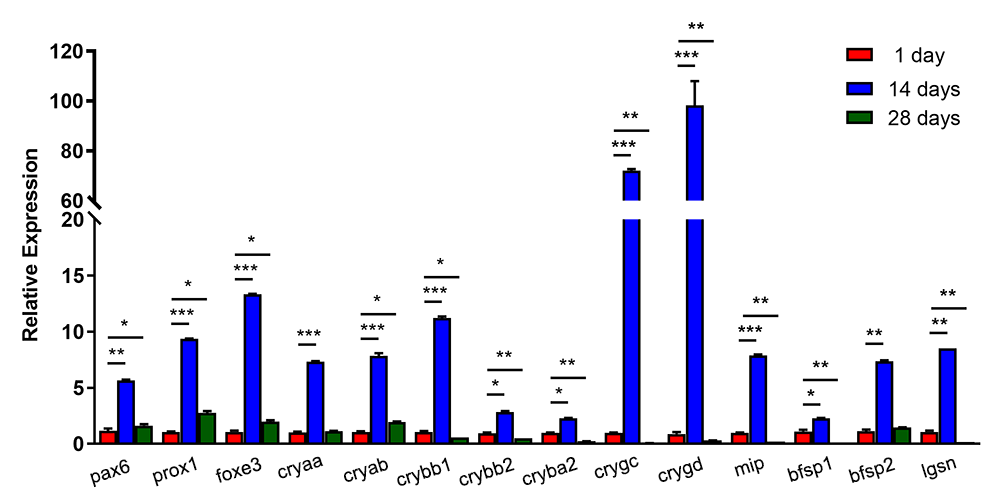

Supplement: Supplementary file 2 — Additional file 2: Fig. S2. The effect of NAM on the in vitro regenerated lens at different time point. [file 13287_2022_2862_MOESM2_ESM.tif]
